# Characterisation of Plant Growth-Promoting Endophytic Bacteria from Sugarcane and Their Antagonistic Activity against *Fusarium moniliforme*

**DOI:** 10.21315/tlsr2021.32.3.6

**Published:** 2021-09-30

**Authors:** Nittaya Pitiwittayakul, Duanpen Wongsorn, Somboon Tanasupawat

**Affiliations:** 1 Department of Agricultural Technology and Environment, Faculty of Sciences and Liberal Arts, Rajamangala University of Technology Isan, Nakhon Ratchasima Campus, Nakhon Ratchasima 30000, Thailand; 2 Department of Biochemistry and Microbiology, Faculty of Pharmaceutical Sciences, Chulalongkorn University, Bangkok 10330, Thailand

**Keywords:** Antifungal Activity, Endophytic Bacteria, *Fusarium moniliforme*, Plant Growth Promoting Bacteria, Sugarcane

## Abstract

The use of endophytic bacteria in agriculture provides an effective way of improving crop yield and significantly reducing chemical usage, such as fungicides. This research was conducted to explore endophytic bacteria with plant growth promotion (PGP) and antifungal activities against *Fusarium moniliforme* AIT01. In this study, we obtained 52 isolates of endophytic bacteria associated with the roots and stems of sugarcane from Nakhon Ratchasima province, Thailand. *In vitro* antagonistic activity test showed that 14 out of 52 isolates had antagonistic activity against the fungal pathogen *F. moniliforme* AIT01. These antagonistic endophytic bacteria were identified as belonging to six different species as follows: *Nguyenibacter vanlangensis*, *Acidomonas methanolica*, *Asaia bogorensis, Tanticharoenia aidae*, *Burkholderia gladioli* and *Bacillus altitudinis* based on phenotypic characteristics, along with phylogenetic analysis of their 16S rRNA gene sequences. Seven isolates effectively inhibited *F. moniliforme* AIT01 mycelial growth by up to 40%. The volatile compounds of six isolates reduced the growth of *F. moniliforme* AIT01 by over 23%. Moreover, riceberry rice seedlings previously treated with *B. gladioli* CP28 were found to strongly reduce infection with phytopathogen by 80% in comparison to the non-treated control. Furthermore, the isolates also showed relevant PGP features, including ammonia production, zinc and phosphate solubilisation, auxin and siderophore biosynthesis. These results demonstrated that the tested endophytic bacteria could be successfully utilised as a source of PGP and biocontrol agent to manage diseases caused by *F. moniliforme*.

**Highlights**

- Fourteen endophytic bacteria belonging to genera *Nguyenibacter, Acidomonas, Asaia, Tanticharoenia, Burkholderia* and *Bacillus* were isolated from roots and stems of sugarcane.
- The bacterial endophytes from sugarcane inhibited the growth of phytopathogen, *Fusarium moniliforme in vitro* and *Burkholderia gladioli* CP28 strongly reduced fungal infection in rice seedlings.
- The tested endophytic bacteria possessed the plant growth-promoting activities such as ammonia production, zinc and phosphate solubilisation, indole acetic acid, and siderophore biosynthesis.

## INTRODUCTION

Endophytic bacteria spend at least one period of their life cycles inside the tissues of plants without inducing any disease symptoms (Hardoim *et al*. 2015). Some of them are known as plant growth-promoting bacteria (PGPB) that play roles in agriculture through direct and indirect mechanisms. The biosynthesis of phytohormones, nitrogen fixation, ACC (1-aminocyclopropane-1-carboxylate) deaminase activity, phosphate or zinc solubilisation, and uptake of iron by siderophore production are the direct mechanisms that lead to promoting plant growth. The indirect mechanisms act as biocontrol and reduce or prevent the deleterious effects caused by phytopathogenic microorganisms. Indirect mechanisms contain antibiotics, ACC deaminase, cell wall degrading enzymes, hydrogen cyanide, siderophores, induced systemic resistance, and quorum quenching (Olanrewaju *et al*. 2017).

In Thailand, sugarcane (*Saccharum officinarum* L.) has long been recognised as one of the most efficient crops for the production of sugar, renewable energy, and biomaterials. The diversity of endophytic bacteria in alpha-, beta-, and gamma-*Proteobacteria* subgroups associated with sugarcane have been reported to have considerable benefits on their development. *Gluconacetobacter diazotrophicus* strain belonging to the family *Acetobacteraceae* was the first recorded nitrogen-fixing endophytic bacteria isolated from sugarcane (Cavalcante & Döbereiner 1988; Gillis *et al*. 1989). *Tanticharoenia aidae* and *Acetobacter sacchari*, were the second and third species, respectively, of plant growth promoting bacteria associated with sugarcane (Vu *et al*. 2016; 2019b). Additionally, various kinds of bacterial endophytes such as *Burkholderia*, *Raoultella*, *Ralstonia*, *Mesorhizobium*, *Ochrobactrum*, *Sphingomonas, Novosphingobium, Pantoea* and *Bacillus* were found in sugarcane (Tam & Diep 2014; Muangthong *et al*. 2015; Luo *et al*. 2016; Rezamahalleh *et al*. 2019; Zeng *et al*. 2020; Rocha *et al*. 2021).

A major chronic problem for crop production and ecosystem stability globally is plant pathogenic microorganisms. *Fusarium* species from the section *Liseola* are among the phytopathogenic fungi which generally infect three crops: sugarcane, rice and maize, even though other species have also been found on these crops (Leslie & Summerell 2006). *Fusarium moniliforme* or *Gibberella fujikuroi* (the perfect stage of *F. moniliforme*) have been reported as the causal agents of Pokkah Boeng in sugarcane. This disease reduced the quality of the harvested crop, particularly among varieties with high sugar yields, which ranged from 40.8% to 64.5% depending on the variety (Vishwakarma *et al*. 2013). Furthermore, *F. moniliforme* was a causative agent of rice Bakanae, a major disease that affects rice seedlings (Ma *et al*. 2008). *F. moniliforme* infected rice seedlings during germination or early stages of growth (Sunder *et al*. 2014). Rice plants may become infected after transplanting, resulting in weak tillering and poor grain filling. The disease, at a later stage, produced a yield loss of 10%–20% and the loss might be higher than 70% at the outbreak of the disease (Ma *et al*. 2008). Biological control is a promising approach that has proved effective in the control of many plant pathogens, including *F. moniliforme* (Hossain *et al*. 2016; Arya *et al*. 2017; Zain *et al*. 2019; Khaskheli *et al*. 2020). The isolation and identification of endophytic bacteria possessing biological mechanisms against phytopathogens is essential for agricultural manipulation.

Most of the research on bacterial endophytes in sugarcane has focused on diazotrophs. There are a few reports that evaluate endophytic bacteria regarding biological control. Thus, the objectives of this study are to isolate and identify the endophytic bacteria isolated from sugarcane that could be used as biocontrol agents against *F. moniliforme*, a causal agent of Pokkah Boeng in sugarcane and Bakanae in rice. Moreover, the antagonistic endophytic bacteria obtained were tested for PGP traits to assess their efficiency in improving plant growth.

## MATERIALS AND METHODS

### Isolation of Bacterial Endophytes

Sugarcane samples were collected from five districts in Nakhon Ratchasima Province, Thailand. The sampling locations included Chaloem Phrakiat, Chakkarat, Huai Thalaeng, Phimai and Chum Phuang. Three individual plants were collected per site. The roots and stems of the samples were rinsed under running tap water to remove attached clay, and then the surface sterilisation was performed. To sterilise, the roots and stems were soaked in 70% ethanol for 3 min, immersed in 2.5% fresh sodium hypochlorite (NaOCl) solution for 5 min, washed with 70% ethanol for 30 sec and followed by rinsing with sterilised water five times. To confirm the disinfection protocol, aliquots of the sterile water used in the final rinse were plated on tryptic soy agar (TSA) medium (HiMedia, India) and the plates were examined for bacterial growth after incubation at 30°C for 3 days. Samples were cut into small pieces (0.5 cm) and ground with a sterile mortar and pestle. The tissue extracts were then serially diluted in sterile 1% sucrose solution. Aliquots (500 μL) were added to semisolid Liquid Glucose Ivo (LGI) or LGI broth (%w/v: sucrose, 10; KH_2_PO_4_, 0.06; K_2_HPO_4_, 0.02; MgSO_4_, 0.02; CaCl_2_, 0.002; FeCl_3_, 0.001; Na_2_MoO_4_, 0.0002) (Cavalcante & Dobereiner 1988; Vu *et al*. 2013) and incubated at 30°C for 4–6 days. When bacterial growth was observed in LGI semisolid and broth as white or yellow particles, the culture was streaked and purified onto LGI agar plates and incubated at 30°C for 6–7 days. Bacterial colonies were differentiated based on colonial appearance and pigmentation.

### Test of Antagonistic Effects

A mycelial plug (5 mm) of the phytopathogen *Fusarium moniliforme* AIT01 was inoculated on one side of a Petri dish containing potato dextrose agar (PDA) at 20 mm away from the periphery, and each endophytic bacterial isolate was streaked as a line onto the medium exactly opposite to the mycelial disc 20 mm away from the periphery. The dual culture plate was incubated at 28°C for 7–14 days. The inhibition zones between the fungal isolate and endophytic bacterial isolates were measured and percent inhibition of fungal pathogen growth was calculated. Five replications were performed for each isolate. Bipartite Petri dishes were used to investigate the inhibitory effect of volatile compounds emitted by endophytic bacteria on the growth of the *F. moniliforme* AIT01. Bacterial isolates were inoculated onto one side of the bipartite petri dish containing PDA, while a 5 mm mycelial mat of *F. moniliforme* AIT01 was transferred to the other side. The plates were parafilm-wrapped and incubated at 28°C and the diameters of the fungal colonies were measured after incubating for 14 days. The inhibition rate of mycelial growth was calculated based on the difference between the treatment and control according to the formula (Saechow *et al*. 2018):


$$\text{Percent Inhibition of Radial Growth }(\text{PIRG})=\frac{R1-R2}{R1}\times 100$$

where, *R*1 is the radial growth of *F. moniliforme* in the control plate, and *R*2 is the radial growth of *F. moniliforme* in the antagonist-tested plate.

### Identification of Plant Growth-Promoting Endophytic Bacteria Associated with Sugarcane

The 14 selected endophytic bacterial strains among 52 isolates were subjected to identification and further study.

#### Phenotypic characterisation

The morphological, cultural, biochemical and physiological characteristics as well as colony and cell morphology, Gram staining, catalase, oxidase, MR-VP, indole production, nitrate reduction, citrate utilisation; acetate and lactate oxidation; lysine and ornithine decarboxylation; hydrolysis of gelatin and L-arginine, and blood haemolysis of each isolate were investigated (Asai *et al*. 1964; Barrow & Feltham 1993). Growth at different temperatures (40°C and 45°C) and different concentrations of NaCl (1%, 3%, and 5% w/v) were determined. Acid production from carbohydrates was examined as described by Tanasupawat *et al*. (1998).

#### Genotypic characterisation

Genomic DNA of endophytic bacteria isolated from sugarcane was directly amplified using colony PCR or by isolation using a genomic isolation kit (Vivantis Technologies Sdn. Bhd., Malaysia). The amplification and sequencing of the 16S rRNA gene were performed with the forward primer 27F (5′-AGAGTTTGATCCTGGCTCAG-3′) and the reverse primer 1525R (5′-AAAGGAGGTGATCCAGCC-3′) (Devereux & Wills 1995). PCR amplification was done as described by Seearunruangchai *et al*. (2004). The purified PCR products were sequenced by the First Base Laboratory (Selangor, Malaysia).

The sequence obtained was analysed and edited by using the Chromas 2.33 and BioEdit programmes (Hall 1999). The 16S rRNA gene sequences of the isolate and all type strains related to the isolate were aligned using CLUSTAL W version 1.83 (Thompson *et al*. 1994). The phylogenetic trees based on the 16S rRNA gene were constructed using neighbour-joining approach in MEGA version 7 software (Saitou & Nei 1987; Kumar *et al*. 2016). Kimura’s two-parameter model was used to calculate the distance between the sequences in neighbour-joining analysis (Kimura 1980). Bootstrap values were estimated based on 1,000 replicates (Felsenstein 1985). The 16S rRNA gene sequence similarities (%) of the isolates to each type strains were obtained from pairwise sequence alignment using EzBioCloud server (http://eztaxon-e.ezbiocloud.net/) (Kim *et al*. 2012).

### Production of Indole-3-Acetic Acid (IAA)

The IAA production was evaluated by a modified colorimetric method (Glickmann & Dessaux 1995). The isolates were cultivated in TSB medium (HiMedia, India) supplemented with 1 μg of L-tryptophan (HiMedia, India) per mL (0.1% tryptophane (w/v)) and incubated at 30°C for 72 h under shaking conditions. The amount of indole compounds produced by each isolate was determined after removing cells by centrifugation at 13,000 rpm for 15 min, at 4°C. Two millilitre of the Salkowski’s reagent (2% 0.5M FeCl_3_, in 35% HClO_4_ solution) were added into the supernatant, mixed and kept in darkness at room temperature for 30 min (Gordon & Weber 1951). IAA production was detected as the development of a pink colour, and its absorbance was measured at 530 nm compared to a standard indole-3-acetic acid solution (Sigma-Aldrich, St. Louis, MO, USA). The experiment was performed in triplicate for each isolate.

### Production of Ammonia

The ammonia production of the isolates was tested by cultivating them in peptone water and incubating them at 30°C for 48 h. The bacterial culture was centrifuged at 4°C for 15 min. The supernatant was mixed with 0.5 mL of Nessler’s reagent. The development of pale yellow to dark brown colour was considered as a positive test for ammonia production (Cappuccino & Sherman 2001).

### Plate Assay for Screening of Siderophore-Producing Strains

Siderophore production was assessed using the universal procedure modified by Schwyn and Neilands (1987). The bacterial isolates were grown in tryptic soy broth (TSB) on a rotary shaker at 150 rpm, at room temperature for 24 h. Then, 10 μL of bacterial suspension was dropped on Chrome azurol S (CAS) agar plates and incubated for 7 days at room temperature. The appearance of a yellowo-range halo zone around the spot was considered as siderophore production.

### Phosphorus and Zinc Solubilising Activity

The qualitative ability of the isolates to solubilise tricalcium phosphate was determined using Pikovskaya medium (PVK agar). The endophytic bacteria were first cultivated in TSB broth and then incubated at 30°C for 24 h. The 10 μL bacterial suspensions cultivated in TSB medium were spotted onto PKV agar and incubated at 30°C for 7 days. A halo zone formation around a bacterial colony indicated phosphate solubilisation. The solubilisation index (SI) was calculated as the ratio of the solubilisation zone diameter to the colony diameter (Pande *et al*. 2017).

Tris-mineral salts medium was used to determine zinc solubilisation potential. Glucose (1% w/v) was selected as the carbon source. The medium was separately supplemented with 0.1% insoluble zinc compounds, viz., zinc carbonate (ZnCO_3_), zinc oxide (ZnO), and zinc phosphate (Zn_3_(PO_4_)_2_). Subsequently, a 10 μL of bacterial suspension cultivated in TSB medium overnight was spot-inoculated onto the agar plate and incubated at 30°C for 7 days. Following incubation, the diameter of halo zone around the colony and bacterial colony was determined and the values were used to calculate the SI (Gandhi & Muralidharan 2016). The SI was determined by formula given as follows:


$$\text{Solubilisation index }(\text{SI})=\frac{\left(\text{Colony diameter}+\text{Halo zone diameter}\right)}{\text{Colony diameter}}$$

### Antifungal Activity of Endophytic Bacterial Strain on Rice

Riceberry rice seeds (*Oryza sativa*) were surface-sterilised with 4% NaOCl in an orbital shaker for 1 h and rinsed with sterile distilled water. Seeds were immersed in a 95% ethanol solution for 5 min. Afterwards, the seeds were washed several times with autoclaved double distilled water (Verma *et al*. 2018). For the infection experiment, surface sterilised seeds were soaked overnight in the endophytic bacterial suspension and then treated with spore suspension of *F. moniliforme* AIT01 (1.1 × 10^6^ spores/mL), along with bacterial free controls for 2 h. Seeds treated with only sterile water were used for negative control. After that, 50 seeds of each treatment were placed onto sterile Petri dishes and incubated for seven days at room temperature. Treatments were set up in triplicate. Seed germination was recorded and the mean germination percentage was calculated. Microscopic observation of seedling roots was performed daily to determine *F. moniliforme* AIT01 infection in root tissues after seven days of fungal inoculation. The presence of fungal mycelium on the surfaces of root tissues indicated fungal infection. The percentages of seedlings infected with *F. moniliforme* AIT01 were calculated according to the formula:


$$\text{Percent seedling infection }(\%)=\frac{\text{Number of infected seedlings}\times 100}{\text{Total number of seedlings}}$$

## RESULTS

### Isolation of Bacterial Endophytes from Root and Stem of Sugarcane

A total of 52 bacteria were isolated from the roots and stems of healthy sugarcane plants collected from Nakhon Ratchasima Province, Thailand. Thirty-one strains and the remaining were obtained from the roots and stems of sugarcane, respectively. Three isolates were Gram-positive and the others were Gram-negative strains. These isolated strains were subjected to antagonistic tests against *Fusarium moniliforme* AIT01.

### Biocontrol Abilities of Endophytic Bacteria against *Fusarium moniliforme*

**Figure 1:**
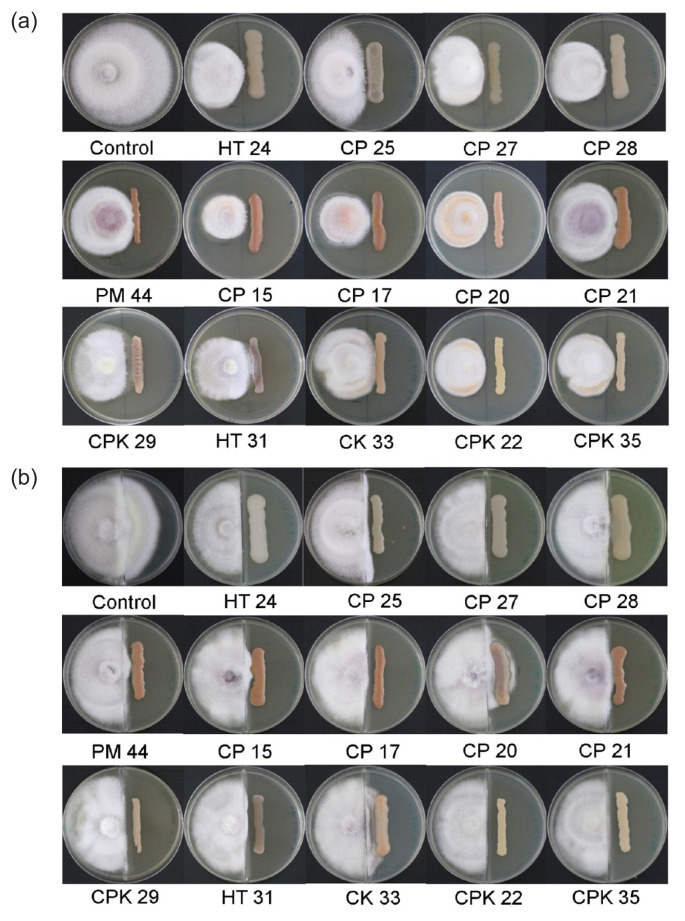
Inhibition of growth of *F. moniliforme* AIT01 by 14 antagonistic endophytic bacterial strains determined by (a) dual culture test, and (b) volatile organic compound inhibition test; on PDA medium. The antifungal activities were measured as the diameter of the zone of inhibition after incubating for 14 days.

A total of 52 isolates were obtained, 14 of which displayed inhibition activity against *F. moniliforme* AIT01 in the initial screenings. All 14 endophytic bacterial isolates were evaluated for antagonistic activities *in vitro* against *F. moniliforme* AIT01 by dual plate techniques and bipartite split-plate growth inhibition assays (volatile compound production) (Table 1, Fig. 1). In dual plate assay, seven isolates, including CP15, CP20, CP21, CPK22, CPK35, CP28 and HT24 showed antagonistic effects on the fungal pathogen *F. moniliforme* AIT01 with PIRG of more than 40%. The isolate CP15 showed the highest antifungal activity with PIRG of 49%. In the volatile antifungal compound assay, most isolates exhibited PIRG at variable levels ranging from 8% to 26% reduction in comparison to control. All tested isolates could produce volatile compounds that inhibited or slowed the growth of *F. moniliforme* AIT01. The isolate CP27 exhibited the highest ability to produce volatile compounds.

### Identification of Endophytic Isolates

**Figure 2:**
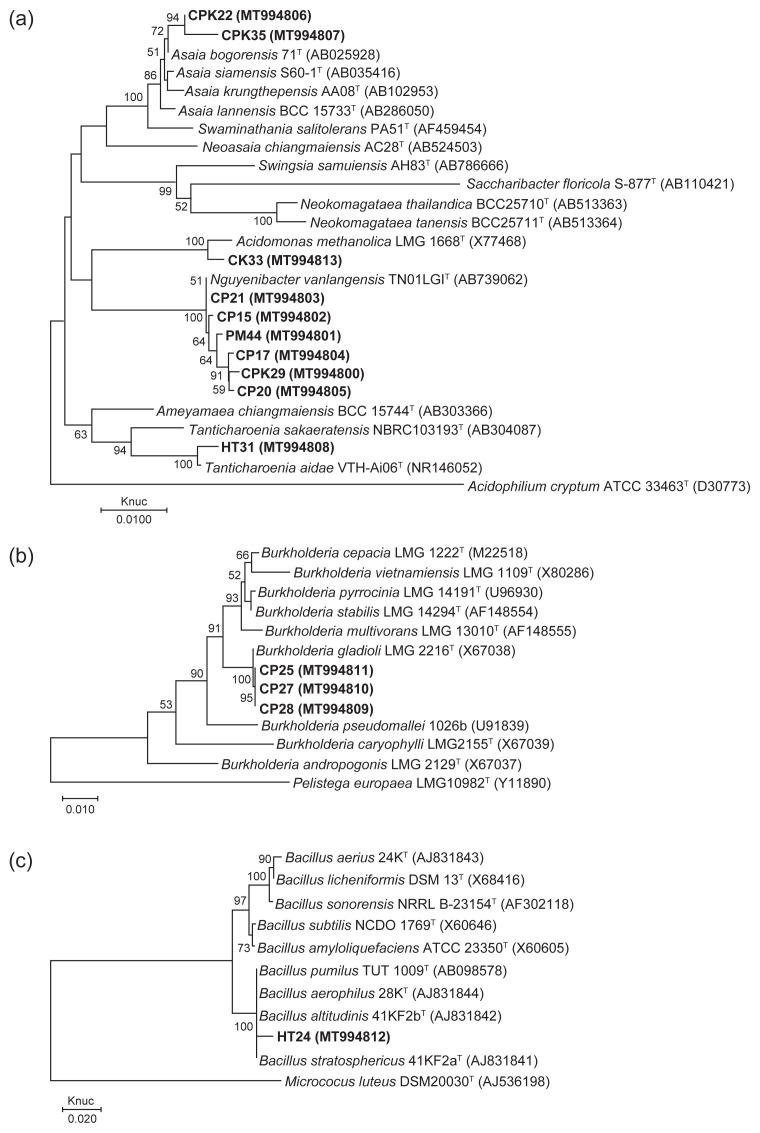
Neighbour-joining tree based on alignment of nucleotide sequences of the 16S rRNA gene of (a) acetic acid bacteria; (b) *Burkholderia;* (c) *Bacillus* isolate and related species. Bootstrap values greater than 50% were indicated. Scale bar represents the number of substitutions per site.

Since 14 strains showed inhibition activities against the fungal pathogen, they were selected for identification and further study. Thirteen Gram-negative and one Gram-positive, rod-shaped bacteria were found in the stems and roots of sugarcane. All the isolates were positive for catalase, lysine and arginine decarboxylation but were negative for indole and MR. Based on the phenotypic characteristics, the 14 isolates could be classified into six groups (Table 2). In a phylogenetic tree based on 16S rRNA gene sequences, all isolates were divided into six groups similar to phenotypic characteristics (Table 3, Fig. 2).

Group I was composed of six isolates. The colonies were creamy-beige, smooth, and convex on GYPG agar. Almost all isolates except CPK29 showed weak growth at 40°C. Only isolates CP15 and CP21 grew weakly in 1% (w/v) NaCl. Water-soluble brown pigment production was observed. Oxidase was variable. They produced acid from only sucrose and oxidised only lactate. On the basis of their 16S rRNA gene sequence and phylogenetic tree analysis (Fig. 2a), isolates CP15, CP17, CP20, CP21 and PM44 showed more than 99% except isolate CPK29 which had 98.93% similarity with *Nguyenibacter vanlangensis* TN01LGI^T^.

Group II consisted of one isolate, CK33. Its colonies were beige to pink, smooth, raised, and entire on GYPG agar. It grew weakly in 1% (w/v) NaCl and at 40°C. The isolate was positive for oxidase. It produced acid from D-mannitol, D-maltose, sucrose, adonitol, raffinose (weakly positive) and lactose (weakly positive). It showed weakly positive lactate oxidation. Based on 16S rRNA gene sequence and phylogenetic analysis (Fig. 2a), isolate CK33 was closely related to *Acidomonas methanolica* LMG 1668^T^ with 100% sequence similarity.

Group III contained 2 isolates, CPK22 and CPK35. The colonies were pink-yellowish white, raised, smooth, and entire on GYPG agar. They grew weakly in 1% (w/v) NaCl and only isolate CPK35 showed weak growth at 40°C. They produced acid from only sucrose and showed positive lactate and acetate oxidation. On the basis of their 16S rRNA gene sequence and phylogenetic tree analysis (Fig. 2a), isolates CPK22 and CPK35 were most closely related to *Asaia bogorensis* 71^T^ with 99.23 and 99.52% sequence similarity, respectively.

Group IV consisted of one isolate, HT31. Its colonies were creamy-beige, smooth, and flat on GYPG agar. It did not grow in 1%, 3% or 5% NaCl and at 40°C. Water-soluble brown pigment production was observed. The isolate was able to produce acid from D-maltose, D-mannitol, D-sorbitol, ducitol, glycerol, ethanol, sucrose, lactose, raffinose and adonitol. It oxidised lactate and acetate. Based on 16S rRNA gene sequence and phylogenetic analysis (Fig. 2a), isolate HT31 was closely related to *Tanticharoenia aidae* VTH-Ai06^T^ with 100% sequence similarity.

Group V was composed of 3 isolates (CP25, CP27, and CP28). The colonies were creamy-white, smooth, opaque, and entire on GYPG agar. They grew in 1 and 3% (w/v) NaCl and at 40°C. Yellow pigment production and α-haemolysis were observed. They were positive for gelatin and aesculin hydrolysis, ornithine decarboxylation, and citrate utilisation. Only isolate CP27 showed weakly positive acid production from sucrose. On the basis of their 16S rRNA gene sequence and phylogenetic tree analysis (Fig. 2b), isolates CP25, CP27, and CP28 were most closely related to *Burkholderia gladioli* LMG 2216^T^ with 99.58%, 99.52%, and 99.67% sequence similarity, respectively.

Group VI consisted of one isolate, HT24. The colonies were creamy-white, flat, and regular margins on GYPG agar. It grew in 1%, 3% and 5% (w/v) NaCl and at 40°C and 45°C. The isolate was positive for VP, gelatin and aesculin hydrolysis, nitrate reduction, and β-haemolysis. It showed weakly positive acid production from sucrose. Based on 16S rRNA gene sequence and phylogenetic analysis (Fig. 2c), isolate HT24 was closely related to *Bacillus altitudinis* 41KF2a ^T^ with 99.19% sequence similarity.

### Characterisation of Potential Plant-Beneficial Traits of Endophytic Bacteria

All 14 isolates were evaluated for PGP activities such as phytohormone, siderophore, ammonia production, zinc and phosphate solubilisation. Table 4 summarises the results of PGP trait evaluation *in vitro*. The IAA producing ability was different among the isolates that were not of the same species. In the presence of tryptophan, the isolated bacteria were able to produce IAA at a concentration of between 2.84 μg/mL and 114.71 μg/mL. The maximum and minimum amounts of IAA were produced by strain CPK22 and CK33, respectively. The bacteria were able to dissolve zinc and phosphate in plates, producing a clear halo around them. Most of the isolates were found to dissolve insoluble tricalcium phosphate (Ca_3_(PO4)_2_) except three isolates, namely HT24, CP28 and CK33. The abilities to solubilise insoluble zinc sources were different among these isolates. All isolates were able to dissolve Zn_3_(PO_4_)_2_. Among 14 isolates, 11 isolates showed zinc solubilisation zones on ZnCO_3_ medium. The isolate CP21 showed a maximum SI of 8.33 and 6.94 on Zn_3_(PO_4_)_2_ and ZnCO_3_ medium, respectively. On ZnO medium, 12 isolates had the ability to dissolve zinc oxide. Isolate CPK35 had the highest SI of 8.33. Among zinc sources, maximum solubilisation by these bacterial isolates was observed in zinc phosphate as compared to other zinc salts. Some isolates did not grow on zinc-supplemented media or solubilise zinc indicating the toxicity of zinc for these isolates. Among the PGP traits screened, it was observed that only four isolates, strains HT24, CP25, CP27 and CP28, were positive for ammonia production. Siderophore production was observed in almost all isolates except CK33 and HT31.

### Efficacy of Antagonistic Endophytic Bacteria Against *F. moniliforme* in Rice Seedling Roots

**Figure 3:**
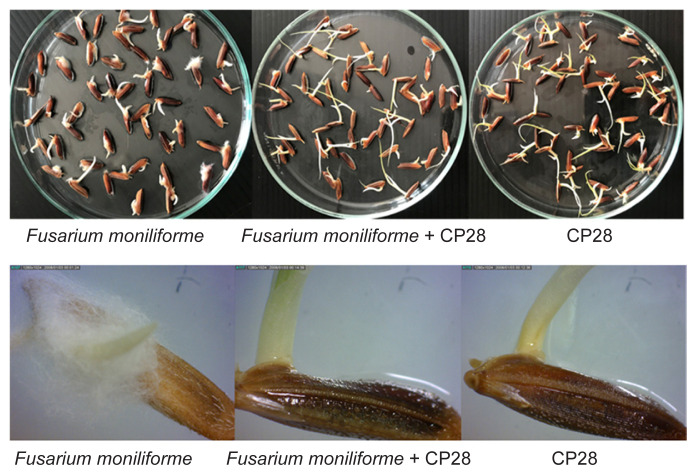
Inoculation of *Burkholderia gladioli* CP28 onto rice seeds protected rice seedlings from *Fusarium moniliforme* AIT01. Rice seeds were treated with sterile water or a suspension of the isolate CP28; with both treated with a suspension of spores of *F. moniliforme*. The growth of fungal mycelium on the surfaces of seeds was observed after incubating at room temperature for 7 days.

In this experiment, the isolate CP28 was selected to test for antifungal activity against *F. moniliforme* on riceberry rice seeds because its antagonistic activities against the fungal pathogen were significantly higher in both dual plate culture and volatile organic compound inhibition tests compared to others. All treatments showed a germination percentage of more than 90%. There was no significant difference between the germination percentage of the seedlings treated with isolate CP28 and those treated with sterile water. The seedlings treated with isolate CP28 were observed to have a reduced infection rate of 9% by *Fusarium* compared to control (90%) (Table 5). The *F. moniliforme* treatment showed significant fungal mycelium growth on the surfaces of rice seeds (Fig. 3).

## DISCUSSION

Currently, the utilisation of PGPB is a potential strategy for improving plant growth and crop productivity in sustainable agriculture. Endophytic microorganisms are considered to be an important bioresource because of their ability to colonise the internal tissues of plants with direct contact (Chaturvedi *et al*. 2016). In the present study, 14 PGPB identified as *Nguyenibacter vanlangensis* (CP15, CP17, CP20, CP21, CPK29 and PM44), *Acidomonas methanolica* (CK33), *Asaia bogorensis* (CPK22 and CPK35), *Tanticharoenia aidae* (HT31), *Burkholderia gladioli* (CP25, CP27 and CP28) and *Bacillus altitudinis* (HT24) were isolated from the roots and stems of sugarcane. *Bacillus* and *Burkholderia* are the most common reported genera of bacterial endophytes (Santoyo *et al*. 2016; Potshangbam *et al*. 2018; Li *et al*. 2019). Some bacterial genera belonging to the *Acetobacteraceae* are known as diazotrophic and are able to promote plant growth by multiple mechanisms (Reis & Teixeira 2015; Vu *et al*. 2019a; 2019b). Most representatives of this group, *Gluconacetobacter*, *Acetobacter*, *Komagataeibacter*, *Swaminathania*, *Asaia*, *Nguyenibacter* and *Tanticharoenia* are commonly associated with the rhizosphere of several plants and also occupy inside roots, leaves and other plant tissues as endophytes (Cavalcante & Döbereiner 1988; Reis & Teixeira 2015; Vu *et al*. 2013; 2016). *In vitro* inhibition of fungal phytopathogens on PDA media against *F. moniliforme* showed that all 14 isolates exhibited PIRG values at variable levels ranging from 16.73% to 49% in comparison to control. Various *Bacillus* species have been reported to inhibit the fungal pathogen *Fusarium* using antimicrobial chemicals and host defense gene induction (Gond *et al*. 2015; Hazarika *et al*. 2019). Many *Burkholderia* species are able to suppress fungal pathogens by various antagonistic mechanisms, such as production of siderophores, antibiotics or lytic enzymes (Tenorio-Salgado *et al*. 2013; Li *et al*. 2019; Ahmad *et al*. 2021). In the case of endophytic bacteria belonging to the *Acetobacteraceae*, there are a few studies on *Gluconacetobacter diazotrophicus* that evaluate endophytic bacteria in relation to biological control. Logeshwarn *et al*. (2011) reported that the biocontrol potential of *G. diazotrophicus* involved pyoluteorin and volatile compound production. Moreover, *G. diazotrophicus* could produce gluconacin, an active bacteriocin against phytopathogenic bacteria (Oliveira *et al*. 2018). In this study, the antagonistic activity of *Nguyenibacter* and *Asaia* might be related to siderophore production. The results from our study indicated that the PGPB strain applied as a seed treatment significantly reduced the percentage of fungal infection. The results demonstrated that bacterial isolate *B. gladioli* CP28 has potent biocontrol activity. Verma *et al*. (2018) also reported that seedlings treated with *Pseudomonas* sp. SY1 had significantly decreased percentage of infection by *Fusarium oxysporum*. Although *B. gladioli* exhibited good biocontrol properties, some strains may be plant-promoters or human, plant and mushroom pathogens (Elshafie & Camele 2021). To use *B. gladioli* for biocontrol applications, its pathogenic potential and antifungal compounds analysis will be assessed in future research.

In addition to the antagonism against pathogenic fungi, all 14 isolates were positive for at least one PGP trait screened. In this study, the isolates CPK22 and CPK35 identified as *Asaia bogorensis* could produce a higher amount of IAA than previously reported strains in the family *Acetobacteraceae* (Madhaiyan *et al*. 2004; Vu *et al*. 2019a). These endophytic bacteria were also established to increase phosphate and zinc solubilisation. Vu *et al*. (2019a) reported that *N. vanlangensis* TN01LGI^T^ and VTH-AC01 were capable of dissolving calcium phosphate as shown in diameters of solubilised halo zones of 17.33 and 15.33 mm, respectively.

## CONCLUSION

Fourteen endophytic bacteria isolated from sugarcane belonged to six species: *Nguyenibacter vanlangensis*, *Acidomonas methanolica*, *Asaia bogorensis, Tanticharoenia aidae*, *Burkholderia gladioli* and *Bacillus altitudinis*, as based on phenotypic characteristics and 16S rRNA gene analyses. All of them exhibited strong antagonistic activities against *Fusarium moniliforme*. *Burkholderia gladioli* CP28 significantly protected rice seedlings from *F. moniliforme* infection. Moreover, plant growth promoting activities such as the production of IAA, siderophores, ammonia and zinc or phosphate solubilisation were reported among these isolates. Therefore, the tested endophytic bacteria are suggested to be useful for controlling plant diseases and stimulating the growth of crop plants.

## Figures and Tables

**Table 1:** Effects of antagonist endophytic bacterial isolates on radial growth of *F. moniliforme* AIT01 in dual culture test and volatile organic compounds inhibition test after

Isolate no.	PIRG (%)	

	Dual culture test	Volatile organic compounds
CP15	49.004 ± 9.18^A^	21.115 ± 0.98^DC^
CP17	16.733 ± 5.79^E^	23.365 ± 0.80^A–D^
CP20	45.085 ± 0.37^AB^	8.467 ± 0.98^E^
CP21	43.161 ± 8.52^AB^	20.900 ± 1.11^DC^
CPK29	28.774 ± 6.31^D^	22.401 ± 0.66^B–D^
PM44	25.498 ± 6.58^D^	19.936 ± 1.60^D^
CK33	33.547 ± 0.48^C–E^	20.043 ± 1.48^D^
CPK22	47.222 ± 3.02^AB^	25.188 ± 1.33^AB^
CPK35	40.385 ± 1.69^A–C^	23.901 ± 0.37^A–C^
HT31	38.197 ± 5.70^BC^	19.614 ± 5.03^D^
CP25	28.166 ± 4.08^D^	22.923 ± 0.48 ^B–D^
CP27	37.821 ± 2.31^B–D^	26.688 ± 2.94^A^
CP28	47.115 ± 0.84^AB^	23.258 ± 2.77^AB^
HT24	42.201 ± 1.29^A–C^	24.009 ± 0.80^A–C^

*Note:* SD = standard deviation. Data in the table are expressed as mean ± SD. Mean with same letters in each column are not significantly different at *P* < 0.05 level by Duncan’s new multiple range test.

**Table 2:** Morphological, physiological and biochemical characteristics of isolates.

Characteristic	Group I						II	III		IV	V			VI
					
	CP15	CP17	CP20	CP21	CPK29	PM44	CK33	CPK22	CPK35	HT31	CP25	CP27	CP28	HT24
Gram staining	−	−	−	−	−	−	−	−	−	−	−	−	−	+
Colony colour*	CB						BP	PY		CB	CW			CW
Soluble pigment*	B	B	B	B	B	B	−	−	−	B	Y	Y	Y	−
Growth at 40°C	w	w	w	w	−	w	w	−	w	w	+	+	w	+
at 45°C	−	−	−	−	−	−	−	−	−	−	w	w	−	w
Growth in 1% NaCl	w	−	−	w	−	−	w	w	w	−	+	+	+	+
in 3% NaCl	−	−	−	−	−	−	−	−	−	−	+	+	+	+
Oxidase	+	+	−	+	+	+	+	−	−	−	−	−	−	−
VP	−	−	−	−	−	−	−	−	−	−	−	−	−	+
Ornithine decarboxylation	−	−	−	−	−	−	−	−	−	−	+	+	+	−
Nitrate reduction	−	−	−	−	−	−	−	−	−	−	−	−	−	+
Lactate oxidation	+	+	+	+	+	+	w	+	+	+	−	−	−	−
Acetate oxidation	−	−	−	−	−	−	−	+	+	+	−	−	−	−
Citrate utilisation	−	−	−	−	−	−	−	−	−	−	+	+	+	−
Aesculin hydrolysis	−	−	−	−	−	−	−	−	−	−	+	+	+	+
Gelatin hydrolysis	−	−	−	−	−	−	−	−	−	−	+	+	+	+
β-Hemolysis	−	−	−	−	−	−	−	−	−	−	−	−	−	+
Acid production from:														
D-Mannitol	−	−	−	−	−	−	+	−	−	+	−	−	−	−
D-Maltose	−	−	−	−	−	−	+	−	−	+	−	−	−	−
D-Sorbitol	−	−	−	−	−	−	−	−	−	+	−	−	−	−
Ducitol	−	−	−	−	−	−	−	−	−	+	−	−	−	−
Glycerol	−	−	−	−	−	−	−	−	−	+	−	−	−	−
Ethanol	−	−	−	−	−	−	−	−	−	+	−	−	−	−
Sucrose	+	+	w	w	w	w	+	+	+	+	−	w	−	w
Lactose	−	−	−	−	−	−	w	−	−	+	−	−	−	−
Raffinose	−	−	−	−	−	−	w	−	−	+	−	−	−	−
Adonitol	−	−	−	−	−	−	+	−	−	+	−	−	−	−

*Note:*

* CB = Creamy-beige; CW = Creamy-white; BP = Beige to pink; PY = Pink-yellowish white; B = Brown; Y = Yellow.

+ = positive reaction; w = weak reaction; − =, negative reaction

**Table 3:** Molecular identification of isolates based on 16S rRNA gene sequencing.

Source	Isolate no.	Group	Accession no.	Nearest relatives	% Similarity	Sequence length (bp)
Root	CP15	I	MT994802	*Nguyenibacter vanlangensis* TN01LGI^T^	99.50	1,419
Root	CP17	I	MT994804	*Nguyenibacter vanlangensis* TN01LGI^T^	100	770
Root	CP20	I	MT994805	*Nguyenibacter vanlangensis* TN01LGI^T^	99.67	1,253
Root	CP21	I	MT994803	*Nguyenibacter vanlangensis* TN01LGI^T^	99.36	1,258
Root	CPK29	I	MT994800	*Nguyenibacter vanlangensis* TN01LGI^T^	98.93	840
Stem	PM44	I	MT994801	*Nguyenibacter vanlangensis* TN01LGI^T^	100	810
Root	CK33	II	MT994813	*Acidomonas methanolica* LMG 1668^T^	100	840
Root	CPK22	III	MT994806	*Asaia bogorensis* 71^T^	99.23	910
Stem	CPK35	III	MT994807	*Asaia bogorensis* 71^T^	99.52	840
Stem	HT31	IV	MT994808	*Tanticharoenia aidae* VTH-Ai06^T^	100	700
Stem	CP25	V	MT994811	*Burkholderia gladioli* LMG 2216^T^	99.58	1,466
Stem	CP27	V	MT994810	*Burkholderia gladioli* LMG 2216^T^	99.52	840
Stem	CP28	V	MT994809	*Burkholderia gladioli* LMG 2216^T^	99.67	910
Root	HT24	VI	MT994812	*Bacillus altitudinis* 41KF2a^T^	99.19	738

**Table 4:** Plant growth promoting traits of isolates.

Isolate no.	Solubilisation index				Siderophore production	IAA production (μg/mL)	NH_4_ production

	Zinc			Phosphate			
	
	ZnO	ZnCO_3_	Zn_3_(PO_4_)_2_	Ca_3_ (PO_4_)_2_			
CP15	6.70	6.21	6.60	1.57	+	23.81±3.69	−
CP17	5.14	6.22	6.80	1.55	+	13.55±0.47	−
CP20	5.85	5.80	6.65	1.52	+	27.00±2.11	−
CP21	8.32	6.94	8.33	1.55	+	14.47±0.53	−
CPK29	6.05	5.80	6.70	1.45	+	22.99±0.88	−
PM44	4.68	5.80	6.65	1.55	+	34.51±3.99	−
CK33	4.39	-	6.33	-	−	2.84±0.02	−
CPK22	5.44	4.71	6.06	1.20	+	114.71±4.99	−
CPK35	8.33	4.88	5.71	1.48	+	89.40±1.04	−
HT31	-	-	7.17	1.42	−	22.84±3.21	−
CP25	2.73	4.00	4.15	1.29	+	4.94±0.17	+
CP27	3.60	5.50	4.10	1.35	+	3.54±0.12	+
CP28	3.90	4.16	3.83	-	+	4.18±0.48	+
HT24	-	-	1.52	-	+	3.45±0.12	+

*Note:* + = positive reaction; − = negative reaction.

**Table 5:** Effect of antagonistic isolate CP28 on riceberry rice seed germination and seedlings infection after 7 days of infection with *F. moniliforme* AIT01.

Treatment	% Infection of seedling	% Germination
*Fusarium moniliforme* AIT01	90 ± 5.29^A^	93 ± 1.15^AB^
*F. moniliforme* AIT01 + CP28	9 ± 1.15^B^	91 ± 1.15^B^
CP28	0^C^	94 ± 2.00^AB^
Sterile water	0^C^	95 ± 2.31^A^

*Note:* SD = standard deviation. Data in the table are expressed as mean ± SD. Mean with same letters in each column are not significantly different at *P* < 0.05 level by Duncan’s new multiple range test.
